# Association of the Osseodensification Technique with Soft Tissue Substitute in a Limitrophe Edentulous Area: A Clinical Case Report with 5-Year Follow-Up

**DOI:** 10.1155/2024/6509451

**Published:** 2024-02-05

**Authors:** André Luiz Berbel de Souza, Rogério Jorqueira dos Reis, Rodrigo Mendes Ferreiro Girondo, Rafaela Pascon, Alexandre Cabrera, Gustavo Vargas da Silva Salomão

**Affiliations:** ^1^Universidade Ibirapuera, Avenida Interlagos 1329, Chácara Flora, São Paulo, SP 04661-100, Brazil; ^2^CTBMF (ACDBS), Av. Marechal Deodoro, 71, Santos, SP CEP 11060-401, Brazil; ^3^São Leopoldo Mandic, Rua Dr. José Rocha Junqueira, 13, Campinas, SP CEP 13045-755, Brazil; ^4^ABO Rio Claro, Av 16, 1768, Rio Claro, SP CEP 13500-460, Brazil; ^5^Department of Restorative Dental Sciences, Division of Prosthodontics, University of Florida College of Dentistry, 1395 Center Dr, Gainesville, FL Zip Code 32610, USA; ^6^School of Dentistry, University of São Paulo-FOUSP, São Paulo, Brazil; ^7^Department of Oral Rehabilitation, Universidade Ibirapuera, Avenida Interlagos 1329, Chácara Flora, São Paulo, SP 04661-100, Brazil

## Abstract

The osseodensification (OD) technique differs from conventional milling for dental implant installation in that it preserves the prepared bone and compacts it toward the apex and lateral walls of the socket, resulting in bone compaction. By enabling autografting, bone expansion, and high implant insertion torques, OD has become an increasingly popular option. The aim of this clinical case report is to demonstrate the predictability of combining OD with guided bone and tissue regeneration (GBR/GTR) techniques for bone expansion in the maxilla with reduced thickness, while avoiding other reconstructive surgeries. The report presents the treatment of a 32-year-old female patient who had cosmetic concerns regarding the anterior maxillary region. The patient was using an adhesive prosthesis with pontic on tooth 13 fixed between teeth 12 and 14. After the case was planned, it was decided that bone expansion in the region would be performed using the OD technique. The implant installation (AR Torque, 3.5 × 11.5 mm, Conexão®) and guided bone regeneration (GBR) were done with the assistance of L-PRF (Stick Bone, associated with L-PRF membrane). Following the osseointegration period, a provisional resin crown was fabricated, and a collagen matrix membrane (Mucoderm®) was used to increase vestibular soft tissue volume and shape the patient's gingival profile. After a period of 120 days, the final crown was created and observed for a span of 5 years. The results showed stability of the case along with maintaining its esthetic and satisfactory function. The use of the osseodensification technique coupled with a connective tissue graft substitute has been anticipated for a long time. It has proven to be an excellent alternative to autogenous grafts.

## 1. Introduction

Throughout the evolution of implantology, various methods have been developed to enhance stability between the alveolar bone and dental implants, which is a crucial factor for treatment success [1]. New techniques have been proposed, researched, and refined with the goal of simplifying surgical procedures and boosting predictability [2].

Several factors are known to ensure successful biomechanical fixation of dental implants in the alveolar bone, including primary and secondary stability, osseointegration [3], implant thread geometry, and type [4]. Another critical factor to consider is the bone milling technique, which can vary, with various techniques proposed to improve bone/implant fixation, particularly in bone types III and IV [5].

The conventional milling technique is widely utilized for cutting and removing bone tissue during drilling in order to create a receptor socket, with the primary objective of facilitating the installation of larger diameter implants in osteotomy spaces that have smaller diameters. This technique enhances the insertion torque [1]. Huwais in 2013 [6] developed the osseodensification (OD) technique by utilizing specialized drills to allow the cut bone to be returned to the apex and lateral walls of the prepared socket. This technique provides improved results compared to traditional techniques. OD allows for autografting to take place on the surface of the entire preparation region during bone compaction, as a result of not removing bone tissue [7].

Studies have shown that osseodensification (OD) improves primary stability, leading to greater bone density compared to conventional techniques [7]. This provides a better implant installation environment for regions with lower bone quality [8] and results in less invasive surgeries and better postoperative recovery [9].

It is widely known that tension between the bone and implant is necessary for bone remodeling to begin [10]. Cases performed using the OD technique resulted in higher tension in the surrounding bone, which facilitated the emergence of microcracks beneficial for bone remodeling [11]. This process is vital for successful osseointegration; however, excessive tension should be avoided as it can surpass normal microcrack formation, which can lead to decrease in the bone/implant connection [10].

A frequent challenge in dental implant rehabilitation is the absence of buccal bone, which can detrimentally impact the esthetic and functional outcomes achieved over the long run. Studies [11] have recently shown that a minimum gap of 2 mm between the buccal bone and the implant is necessary for enhanced predictability in this form of treatment.

Bearing in mind that in implant rehabilitation, attention should be paid to the various previously mentioned points while avoiding excessive interventions; the objective of this clinical case report was to demonstrate to the implant dentist the predictability of associating OD with guided bone and tissue regeneration (GBR/GTR) techniques for bone expansion in the maxilla with reduced thickness, thus avoiding other reconstructive surgeries.

## 2. Case Presentation

This case report follows the clinical case reporting guideline (CARE) [12], and the patient provided written informed consent for publication of this case report.

**Figure 1:**
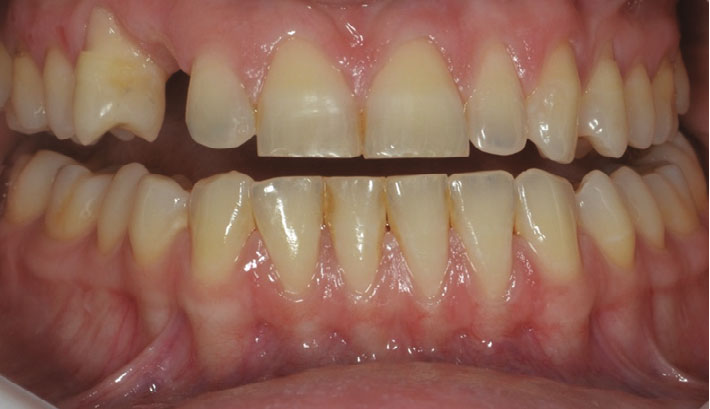
Initial clinical frontal image of the case.

**Figure 2:**
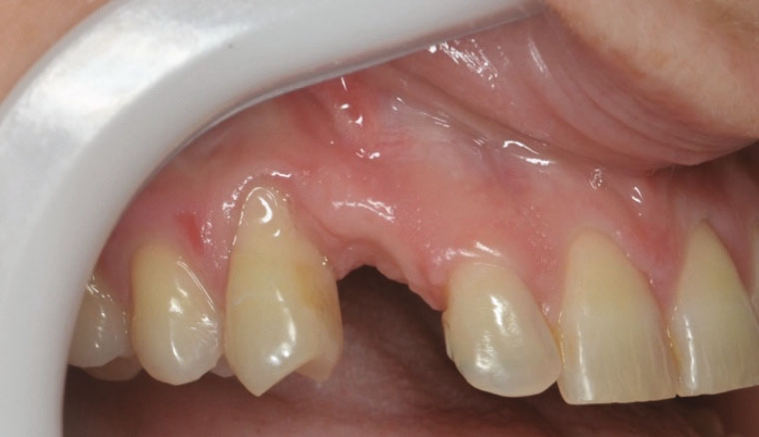
Initial clinical lateral image of the case.

A 32-year-old woman sought dental care at a private clinic in Itatiba, SP, with esthetic concerns in the anterior region of her maxilla. The patient utilized an adhesive prosthesis for tooth 13, affixed to teeth 12 and 14, which had become loose at the time of assessment (Figures 1 and 2). Upon examination, the patient revealed that the dental professional had previously pursued implant surgery in the area when the adhesive prosthesis was initially placed. However, there was insufficient bone for the procedure to proceed as planned.

**Figure 3:**
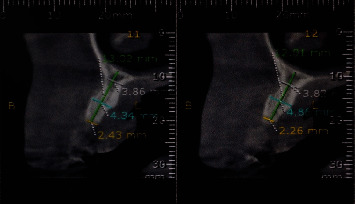
Tomographic section of element 13. It is noted that the thickness is insufficient to install the implant in an adequate position.

After analyzing the computed tomography and clinical examination, it was observed that the thickness of the alveolar bone was inadequate (Figure 3), and there was a reduction in the volume of vestibular soft tissue. Therefore, the proposed planning was to carry out a bone expansion during the first surgical stage, followed by guided bone regeneration aimed at ensuring that the implant is at least 2 mm away from the vestibular wall [13]. In the second surgical stage, guided tissue regeneration was carried out using a collagen matrix membrane (Mucoderm®) [14].

Before the surgery, the patient received orally administered amoxicillin 2 g and Decadron 4 mg one hour prior to the surgical procedure. Intraoral asepsis was performed with 5 ml of 0.12% aqueous chlorhexidine, and extraoral asepsis was performed with 2% aqueous chlorhexidine before the surgical procedure. The injection of local anesthesia was 4% articaine hydrochloride with epinephrine 1 : 100,000 (Articaine 100-DFL®) administered through the terminal infiltrative technique in the designated area.

**Figure 4:**
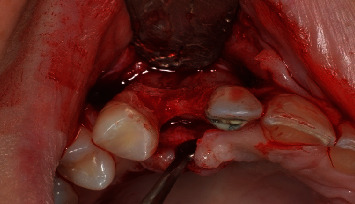
Flap created before the start of the osseodensification technique. Note the buccal bone bulging.

**Figure 5:**
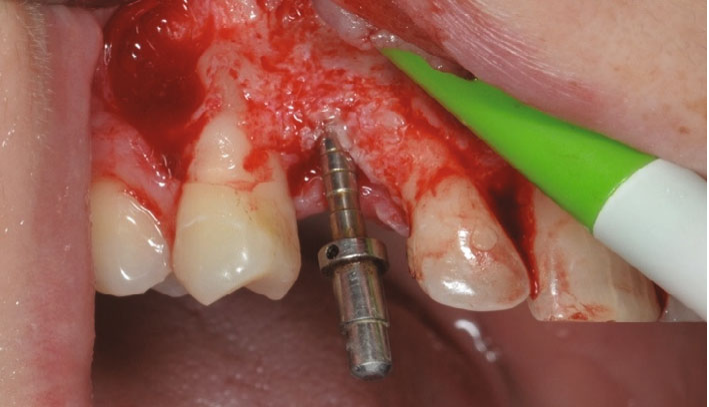
Three-dimensional assessment of surgical preparation.

**Figure 6:**
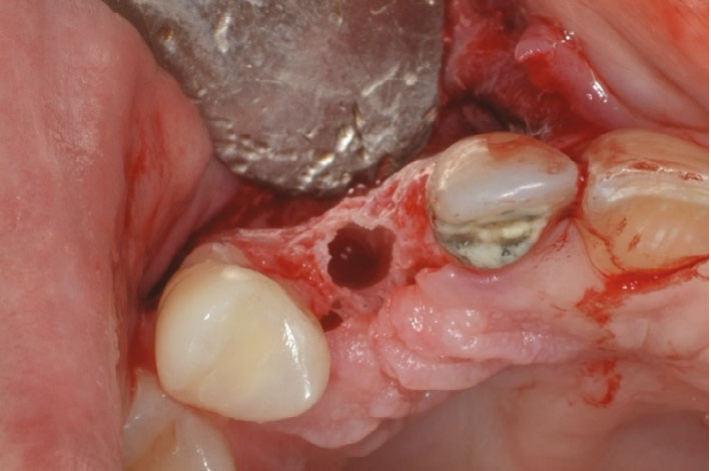
Surgical aspect of the osseodensification technique after the second milling session.

**Figure 7:**
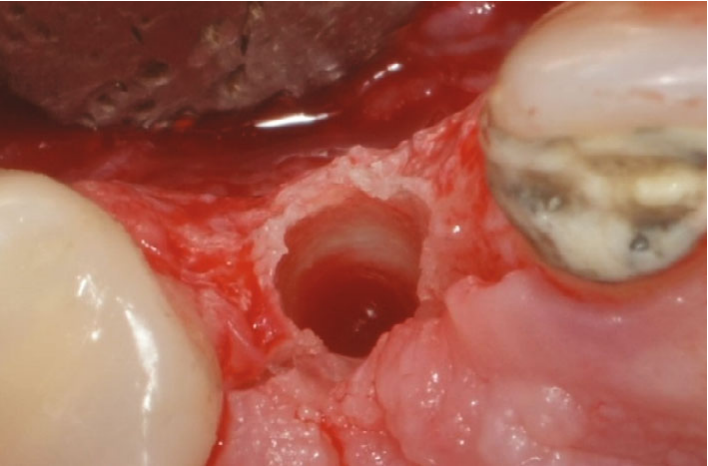
Surgical aspect after the osseodensification technique. Note the characteristic of the condensed bone against the walls of the preparation.

A scalpel was employed to execute an incision at the central margin of element 13, accompanied by relieving sulcular incisions, resulting in the propagation of the flap along the buccal and palatal areas (Figure 4). The OD technique was used in the region with a broad view, employing Versah Burs drills. Initially, the pilot drill was utilized solely to rupture the cortical bone in a clockwise rotation with 800 rpm and ample irrigation. After this stage, the parallelometer was utilized to evaluate the three-dimensional position of the preparation (Figure 5). Drills 1.8, 2.3, and 3.0 were utilized in a counterclockwise direction at a speed of 1200 rpm with plentiful irrigation and at a depth of 11.5 mm. The objective was to compress the alveolar bone against the walls to expand the surgical socket instead (Figures 6 and 7).

**Figure 8:**
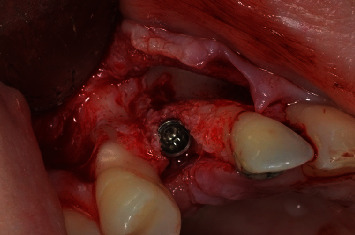
Implant installed 2 mm infra bone, with primary stability of 45 N/cm.

After completing the surgical preparation, the Conexão® implant Morse cone (AR Torque), measuring 3.5 mm in diameter and 11.5 mm in height, was inserted using a surgical contra-angle. The implant was secured to achieve a torque of 20 N/cm with the aid of a ratchet and finished at a position 2 mm below the bone surface with a torque of 45 N/cm, in accordance with the initial plan (Figure 8). Finally, a 2 mm cover screw was placed at the end of the implant procedure.

**Figure 9:**
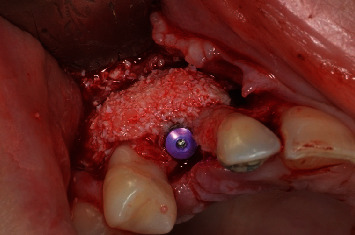
Guided bone regeneration (Stick Bone), to increase bone volume.

**Figure 10:**
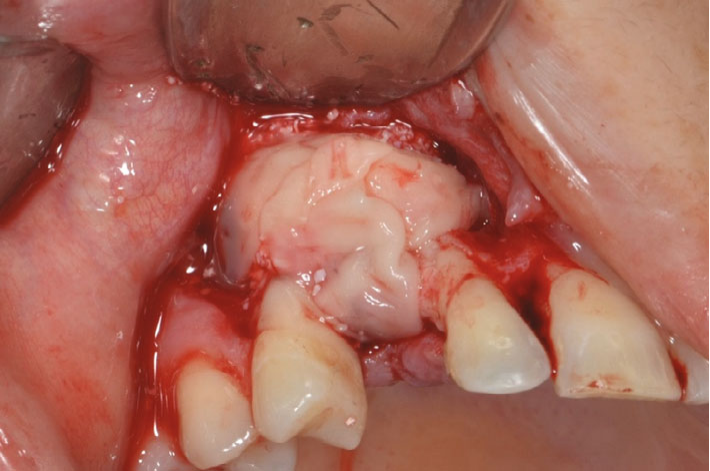
Lateral view of the installation of L-PRF membranes.

**Figure 11:**
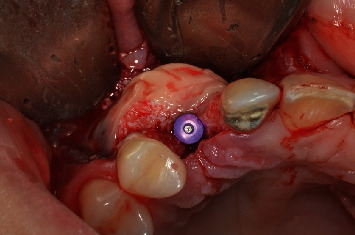
Occlusal view of the installation of L-PRF membranes. It is noted that this involved all the graft material and the implant.

After implant placement, guided bone regeneration (GBR) was performed using the Stick Bone technique along with the leukocyte and platelet-rich fibrin (L-PRF) membrane [15]. The initial step involved blood collection from the patient, followed by a combination of Bio-Oss® and fibrin membrane [16]. The membrane was obtained through vitropressure applied to the collected and centrifuged blood material, resulting in bone substitute hydration and cluster formation [15]. The vestibular region was decorticalized via small perforations using a 1 mm carbide spherical drill (Angelus®) to nourish the graft material. Subsequently, the Stick Bone was positioned along the vestibular wall to increase bone volume (Figure 9). The Choukroun technique [17] was employed, and the resulting membranes were superimposed onto the graft and implant materials to provide protection (Figures 10 and 11).

**Figure 12:**
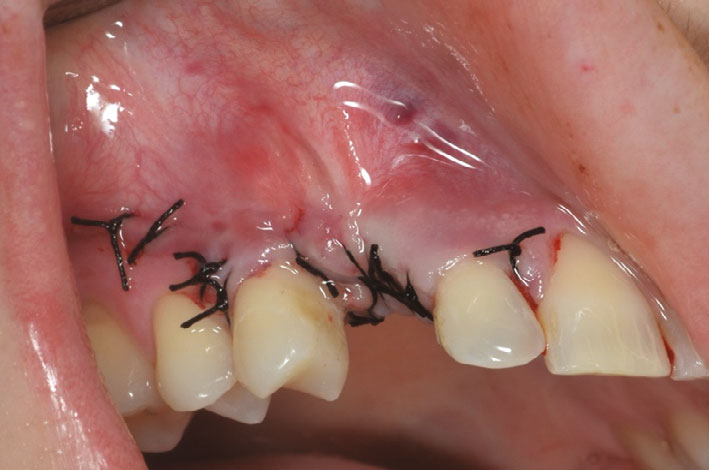
Closure of the surgical wound in first intention. It is noted that the flap was closed without tension, an important factor to avoid suture dehiscence.

The flap was repositioned in a coronal direction over the membranes to begin suturing. To ensure the success of GBR, closing the flap in the first intention was crucial. To achieve this, it was imperative to separate the periosteum under the flap using a 15C scalpel blade to release the tissue [18]. After ensuring that the tissue was free from tension, we used silk threads (Ethicon 4.0, needle 17 cm, and reverse cut 3/8, Johnson & Johnson®) to perform a suture with simple stitches, coapting the borderlines in the first intention (Figure 12).

Following the procedure, the patient was directed to take amoxicillin 500 mg every 8 hours for 7 days, Decadron 4 mg every 12 hours for 2 days, and paracetamol 750 mg every 6 hours for 3 days. Ten days after the surgical procedure, the stitches were removed, and good tissue healing was observed clinically.

**Figure 13:**
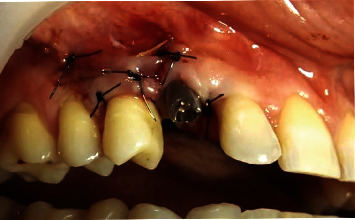
Immediate soft tissue stability was obtained after guided tissue regeneration within the sutures. Note the difference between resorbable and nonresorbable sutures and the characteristics of the provisional post before receiving the immediate crown. The crown was removed temporarily to analyze the characteristics of the provisional abutment.

After completing the required 90-day osseointegration period for the implant [19], the patient progressed to the prosthetic phase of treatment. During this stage, the implant was surgically reopened under anesthesia in the area of element 13 (4% articaine hydrochloride with epinephrine 1 : 100,000, Articaine 100-DFL®). After administering anesthesia, a 15C scalpel blade was used to make an incision over the implant area. Following the location of the cover screw, it was removed using the 0.9 key from the Conexão® prosthetic kit. A temporary abutment from Conexão® was then placed on the implant, and a faceted stock tooth was captured using flow resin. After the provisional was polished and completed, the crown was affixed to the implant using a 20 N/cm torque as per planning. To gain soft tissue vestibular volume without invasive patient surgery, a type I/III natural collagen matrix membrane derived from porcine dermis (Mucoderm®) was installed subsequent to the provisional crown installation. The area where the temporary crown was installed was opened up, using the same local anesthetic approach, followed by a relaxing incision made at the distal region of element 14, extending to the distal region of element 12. Mucoderm® was then positioned after flap detachment and hydrated in saline solution for 30 minutes. For stabilization, it was sutured with reabsorbable thread (Vicryl 4.0, 17 cm needle, and 3/8 reverse cut, Johnson & Johnson®) in a simple stitch. After securing the membrane over the flap, it was moved coronally and sutured according to the manufacturer's instructions [14], with nonresorbable stitches being removed within 10 days (Figure 13). The patient reported no postoperative symptoms.

Four months after provisional crown installation, the definitive crown was created. A 3.5 × 6 mm abutment from Conexão® company designed for a cemented prosthesis was installed with a torque of 32 N. A provisional crown was then relined over it. Following this, open-mold transfer molding was performed. Silicone was used to make the mold in a single step. After preparing the infrastructure, the metal coping was tested before the porcelain was applied. Lastly, the porcelain was taken for testing. After confirming its adequacy, the piece was glazed and installed beneath the abutment using zinc phosphate cementation.

**Figure 14:**
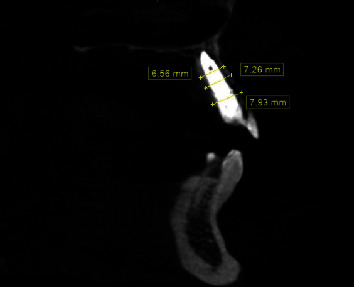
Computed tomography after 5 years of follow-up. It is noted that the buccal and palatal walls were maintained after expansion.

**Figure 15:**
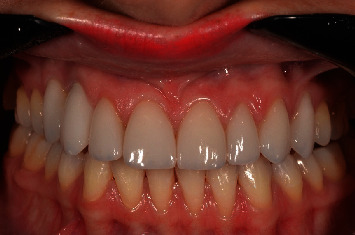
Frontal view of the stability of crown in position after 5 years of follow-up. Note the stability of the peri-implant soft tissue.

**Figure 16:**
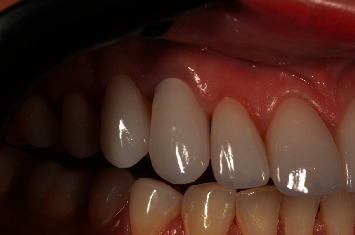
Lateral view of the stability of crown in position after 5 years of follow-up. Note the stability of the peri-implant soft tissue.

**Figure 17:**
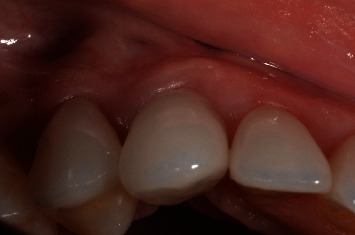
Occlusal view of the stability of crown in position after 5 years of follow-up. Note the stability of the peri-implant soft tissue.

Annual follow-up procedures were carried out upon case completion. In the fifth year of follow-up, a computed tomography scan was requested to evaluate bone volume in the vestibular region. Upon analysis of the tomographic examination, it was observed that the implant remained completely encased by bone tissue with vestibular and lingual surrounding walls preserved (Figure 14). Figures 15–17 depict soft tissue stability 5 years after the follow-up.

## 3. Discussion

High primary stability has been shown to be significant according to the literature [10]. The osseodensification technique enables greater predictability of primary stability, leading to higher insertion torques during implant installation [7]. This can be achieved without prior grafts, resulting in more conservative treatments [20]. The OD technique differs from the conventional technique in that it does not extract bone tissue. Instead, it compresses and grafts particles of the bone tissue, which gives the implant greater stability. This is achieved by employing a hydrodynamic bone preparation, where the serum creates pressure through the movement of the medullary bone portion. As a result, this causes changes in its density, and the desired expansion of the tissue occurs [1–7]. In the 2017 study conducted by Huwais and Meyer [2], the OD technique resulted in increased implant insertion torque. This differs from the average primary stability of 25 N/cm found in literature surrounding the conventional technique. The OD technique demonstrated an ability to generate an average torque of 45 N/cm, deemed the gold standard, and enable immediate loading [2].

Regarding bone expansion conducted through osseodensification, studies like the one conducted by Comuzzi et al. in 2020 [4] exhibit that this technique facilitates predictable expansion of alveolar bone and enhances implant insertion torque in contrast to bone expansion with conventional expanders [21]. These findings support the aforementioned case report, demonstrating bone expansion throughout all thirds of the operative region (Table 1). In the presented case, the implant could not have been installed in the ideal position due to the limited bone. However, by using the osseodensification technique, which condenses the bone on the walls of the alveolus without removing them, it was possible to achieve the appropriate bone thickness for implant placement. This facilitated the optimal placement of the implant without necessitating invasive bone grafting procedures, such as autogenous blocks, which are considered the gold standard for this type of case [22]. This phenomenon arises due to osteodistraction permitting the compression of the alveolar bone against the lateral walls, thereby promoting both expansion and condensation of the bone tissue, leading to increased insertion torque [2, 10–14].

Despite the favorable outcomes reported in this case study, clinical examination remains crucial when planning for perforations. The choice of osseodensification technique is directly influenced by the bone type, surgical objective, and region of operation. This is because the milling cutters in this system can be used in two ways: densifying (counterclockwise) or cutting (clockwise) [2]. It is important to note that the aforementioned factors play a significant role in the selection process.

The effective management of peri-implant tissue was a crucial issue in this clinical case. To ensure positive outcomes, recent studies [23, 24] highlight the significance of having keratinized tissue around the implant for long-term success, resulting in satisfactory pink esthetics at the site [4]. The autogenous connective tissue graft technique represents the gold standard for soft tissue gain, despite its known drawbacks such as a painful postoperative period at the donor site and the requirement for a highly skilled surgeon due to the technique's high surgical complexity [25]. To decrease the considerable morbidity associated with this procedure, we opted for the use of a collagen matrix membrane derived from pigs, which has shown to yield satisfactory and stable outcomes for up to a year [25]. Comparable findings were reported by Papi and Pompa [26], in which the soft tissue remained stable for a period of 12 months, thus indicating the practicality of substituting with this type of material.

What sets this study apart is the application of a combination of techniques for regenerating hard and soft tissue, specifically OD and the utilization of Mucoderm®, with a follow-up period of 5 years. These isolated techniques enable more conservative procedures without the necessity of autogenous grafts, which were the only feasible option previously [27]. Using both techniques in this clinical case at appropriate times yielded predictable and stable results, with reduced morbidity. These findings support current literature [28–30], making it a viable alternative for complex cases.

## 4. Conclusion

Based on the presented clinical case report, it can be concluded that osseodensification, when used in combination with a xenogeneic matrix as a replacement for connective tissue grafts, provides a feasible and durable alternative to autogenous grafts in cases where it is appropriately scheduled and indicated.

## Figures and Tables

**Table 1:** Bone volume before and after the procedure, divided into thirds.

	Average bone volume preoperative (measured in mm)	Average bone volume postoperative (measured in mm)
Apical third of the implant	3.82	6.56
Middle third of the implant	4.86	7.26
Cervical third of the implant	2.26	7.93

Measurements in millimeters.

## Data Availability

The data is available upon corresponding author's request.

## References

[1] Trisi P., Perfetti G., Baldoni E., Berardi D., Colagiovanni M., Scogna G. (2009). Implant micromotion is related to peak insertion torque and bone density. *Clinical Oral Implants Research*.

[2] Huwais S., Meyer E. G. (2017). A novel osseous densification approach in implant osteotomy preparation to increase biomechanical primary stability, bone mineral density, and bone-to-implant contact. *The International Journal of Oral & Maxillofacial Implants*.

[3] Yamaguchi Y., Shiota M., Munakata M., Kasugai S., Ozeki M. (2015). Effect of implant design on primary stability using torque-time curves in artificial bone. *International Journal of Implant Dentistry*.

[4] Comuzzi L., Tumedei M., Piattelli A., Iezzi G. (2020). Osseodensification drilling vs. standard protocol of implant site preparation: an in vitro study on polyurethane foam sheets. *Prosthesis*.

[5] Galli S., Jimbo R., Tovar N. (2015). The effect of osteotomy dimension on osseointegration to resorbable media-treated implants: a study in the sheep. *Journal of Biomaterials Applications*.

[6] Huwais S. (2013). Inventor; Fluted osteotome and surgical method for use.

[7] Bergamo E. T. P., Zahoui A., Barrera R. B. (2021). Osseodensification effect on implants primary and secondary stability: multicenter controlled clinical trial. *Clinical Implant Dentistry and Related Research*.

[8] Witek L., Neiva R., Alifarag A. (2019). Absence of healing impairment in osteotomies prepared via osseodensification drilling. *The International Journal of Periodontics & Restorative Dentistry*.

[9] Trisi P., Berardini M., Falco A., Podaliri V. M. (2016). New osseodensification implant site preparation method to increase bone density in low-density bone: in vivo evaluation in sheep. *Implant Dentistry*.

[10] Coelho P. G., Jimbo R. (2014). Osseointegration of metallic devices: current trends based on implant hardware design. *Archives of Biochemistry and Biophysics*.

[11] Araújo M. G., Silva C. O., Misawa M., Sukekava F. (2015). Alveolar socket healing: what can we learn?. *Periodontology 2000*.

[12] Gagnier J. J., Kienle G., Altman D. G. (2013). The CARE guidelines: consensus-based clinical case reporting guideline development. *Global Advances in Health and Medicine*.

[13] Amaral Valladão C. A., Freitas Monteiro M., Joly J. C. (2020). Guided bone regeneration in staged vertical and horizontal bone augmentation using platelet-rich fibrin associated with bone grafts: a retrospective clinical study. *International Journal of Implant Dentistry*.

[14] Starch-Jensen T., Becktor J. P. (2019). Maxillary alveolar ridge expansion with split-crest technique compared with lateral ridge augmentation with autogenous bone block graft: a systematic review. *Journal of Oral & Maxillofacial Research*.

[15] Miron R. J., Zucchelli G., Pikos M. A. (2017). Use of platelet-rich fibrin in regenerative dentistry: a systematic review. *Clinical Oral Investigations*.

[16] Branemark P. I., Zarb H. A., Albrektsson T. (1985). *Tissueintegrated Prostheses. Osseointegration in Clinical Dentistry*.

[17] Dohan Ehrenfest D. M. (2010). How to optimize the preparation of leukocyte- and platelet-rich fibrin (L-PRF, Choukroun's technique) clots and membranes: introducing the PRF box. *Oral Surgery, Oral Medicine, Oral Pathology, Oral Radiology, and Endodontics*.

[18] Neiva B. T., Neiva G. F., Neiva R. (2022). Lasso guided bone regeneration technique for the management of implant fenestration defects. *The Compendium of Continuing Education in Dentistry*.

[19] Lioubavina-Hack N., Lang N. P., Karring T. (2006). Significance of primary stability for osseointegration of dental implants. *Clinical Oral Implants Research*.

[20] Lahens B., Neiva R., Tovar N. (2016). Biomechanical and histologic basis of osseodensification drilling for endosteal implant placement in low density bone. An experimental study in sheep. *Journal of the Mechanical Behavior of Biomedical Materials*.

[21] Zitzmann N. U., Schärer P. (1998). Sinus elevation procedures in the resorbed posterior maxilla: comparison of the crestal and lateral approaches. *Oral Surgery, Oral Medicine, Oral Pathology, Oral Radiology, and Endodontics*.

[22] de Azambuja Carvalho P. H., Dos Santos Trento G., Moura L. B., Cunha G., Gabrielli M. A. C., Pereira-Filho V. A. (2019). Horizontal ridge augmentation using xenogenous bone graft-systematic review. *Oral and Maxillofacial Surgery*.

[23] Fischer K. R., Scaini R., Chackartchi T., Solderer A., Schmidlin P. R., Testori T. (2023). Soft tissue-related complications around anterior implants: commentary and clinical checklist. *Quintessence International*.

[24] Bressan E., Guazzo R., Tomasi C. (2023). Influence of soft tissue thickness on marginal bone level around dental implants: a systematic review with meta-analysis and trial-sequential analysis. *Clinical Oral Implants Research*.

[25] Zuhr O., Bäumer D., Hürzeler M. (2014). The addition of soft tissue replacement grafts in plastic periodontal and implant surgery: critical elements in design and execution. *Journal of Clinical Periodontology*.

[26] Papi P., Pompa G. (2018). The use of a novel porcine derived acellular dermal matrix (Mucoderm) in peri-implant soft tissue augmentation: preliminary results of a prospective pilot cohort study. *BioMed Research International*.

[27] Fathiazar A., Shariatmadar Ahmadi R., Sayar F. (2022). A comparison between Mucoderm® and connective tissue graft for root coverage. *Journal of Dentistry*.

[28] Mercier F., Bartala M., Ella B. (2022). Evaluation of the osseodensification technique in implant primary stability: study on cadavers. *The International Journal of Oral & Maxillofacial Implants*.

[29] Pai U. Y., Rodrigues S. J., Talreja K. S., Mundathaje M. (2018). Osseodensification - a novel approach in implant dentistry. *The Journal of the Indian Prosthodontic Society*.

[30] Padhye N. M., Padhye A. M., Bhatavadekar N. B. (2020). Osseodensification -- a systematic review and qualitative analysis of published literature. *Journal of Oral Biology and Craniofacial Research*.

